# Development and initial validation of a cutaneous leishmaniasis impact questionnaire

**DOI:** 10.1371/journal.pone.0203378

**Published:** 2018-08-30

**Authors:** Endi Lanza Galvão, Mariana Junqueira Pedras, Gláucia Fernandes Cota, Taynãna César Simões, Ana Rabello

**Affiliations:** Pesquisa Clínica e Políticas Públicas em Doenças Infecto-Parasitárias–Instituto René Rachou—Fundação Oswaldo Cruz, Fiocruz, Belo Horizonte, Minas Gerais, Brazil; Institute of Tropical Medicine Antwerp, BELGIUM

## Abstract

**Background:**

The impact of cutaneous leishmaniasis (CL) on the quality of life of patients has been neglected in research studies worldwide. The few reported studies have used non-specific questionnaires for the disease, which represents a limitation since generic instruments may not address specific aspects of the disease, compromising the evaluation of its real impact. The aim of this paper is to describe the development and the initial validation of an instrument for evaluating the impact of CL, named the Cutaneous Leishmaniasis Impact Questionnaire.

**Methodology:**

The formulation and validation of the instrument consisted of the following steps: (1) literature review; (2) conceptual framework construction and initial item generation; (3) tool analysis by health professionals (*experts*); (4) tool evaluation performed by the patients; and (5) a pilot study with 100 patients with localized CL, evaluated at a reference ambulatory facility in Belo Horizonte, in the state of Minas Gerais, Brazil. The structure of the proposed instrument was analyzed using hierarchical cluster analysis (ICLUST).

**Results:**

Twenty-seven items were initially proposed by the researchers to compose the questionnaire. Content validity (evaluates if the instrument fully assesses the construct of interest) was evaluated by the panel of experts, while face validity (evaluates how potential participants interpret the items) was evaluated by the target population. In this step, some items were excluded, reformulated and/or included. After evaluating a factorial structure of the items in accordance with the cluster analysis, we assembled a questionnaire with 25 items (alpha = 0.86), with high reliability and homogeneity, which address the following: 1) the general impact of the disease (alpha = 0.91, beta = 0.67) and 2) the evaluation of the perception about the treatment and health services (alpha = 0.72, beta = 0.51).

**Conclusions:**

The Cutaneous Leishmaniasis Impact Questionnaire, developed with contributions from patients and *experts*, was confirmed, in this first validation, as a useful and reliable instrument.

## Introduction

Cutaneous leishmaniasis (CL) is a chronic and non-fatal infectious disease that can substantially decrease the physical [1,2], psychological [3] and social [4] quality of life. Skin lesions are the hallmark of CL and are usually found on exposed parts of the body, such as the legs, arms and face [5,6]. Either by the activity of the lesions or the healing process, CL can cause deformities and marked aesthetic damage. The disease affects people living in low-income countries, and the impact on patients and their families also includes substantial economic losses [7,8].

The concept of quality of life (Qol) is increasingly recognized as an important health outcome, based on the individual's perception of his state of health. Questionnaires that evaluate quality of life are instruments that allow the comparison of the full state of health, both individually and collectively, in research or clinical practice. The most commonly used and well-studied life impact tool for CL is the Dermatology Life Quality Index (DLQI) questionnaire [1,2,9–11], designed to measure the impact of general skin disease on a patient’s quality of life during the past week [12]. Although it has been tested for more than 120 different diseases, it was not developed with CL patient input and may not fully capture issues that are unique to these patients.

The lack of a specific tool for evaluating the impact of CL has already been noted as a limitation of previous studies evaluating the quality of life of patients with CL [10]. Among other reasons, the assessment performed through the DLQI, based only on the evaluation of the week prior to the application of the questionnaire, does not allow the distinction between the impact of the disease and the impact of the various treatment options [2]. Notably, an instrument to assess outcomes specifically for CL would be useful for the development of clinical evaluation and to help standardize future research.

The purpose of this paper was to describe the development and validation of a specific questionnaire to assess the impact of CL, including the social, physical, occupational, economic and emotional aspects.

## Methods

The study protocol was reviewed and approved by the Ethics Committee on Human Research of the René Rachou Institute, *Fundação Oswaldo Cruz* [1.337.731], and participants signed informed consent forms prior to study participation.

The questionnaire was developed following five steps: (1) literature review; (2) conceptual framework construction and initial item generation; (3) tool analysis by health professionals *(experts)*; (4) tool evaluation performed by the patients; and (5) completion of a pilot study. Steps 1 and 2 involved mainly qualitative analysis, whereas the last three phases used both qualitative and quantitative methods.

### Literature review

As the first step, a literature review was conducted to search available tools developed to assess the life impact of CL. The search was implemented using the PubMed (MEDLINE), VHL (LILACS and IBECS) and Web of Science databases, in May 2015, without language restrictions and using the combination of “Leishmaniasis”, “Tegumentary Leishmaniasis”, and “Cutaneous Leishmaniasis”, as well as the following text words: “Quality of Life”, “Questionnaire”, “Measurement” and “Health Economic Evaluation”. Although some information could be found about applying pre-existing questionnaires to assess the impact of CL, no instrument specifically developed for CL was identified. All recovered studies were read in full, focusing on the scope of the questionnaire and confirming that many relevant questions were not included in these tools. In summary, this step was used to guide the conceptual framework construction and initial item generation.

### Conceptual framework construction and initial item generation

The aim of this step was to identify the relevant issues for the patients affected by CL, to create the overall initial structure and initial shape of the instrument. A comprehensive review of existing dermatological Qol questionnaires (DLQI, Psoriasis Disability Index and Skindex) and other tools (WHOQOL and the WHOQOL Brief, SF36, EQ-5D, and Work Limitation Questionnaire) was performed to identify an appropriate conceptual framework. Using grounded theory, concepts about the disease related to the global impact perception, as well as physical symptoms, emotional, occupational and economic impact, social relationships, treatment satisfaction and access to health services, were included as important aspects to be evaluated by the new instrument. At least one item of the questionnaire was developed for each issue or concept. Thus, the first questionnaire version emerged from a set of items extracted from existing tools developed for other diseases added to the researcher’s inputs. At the end of this step, a list of 27 potential items addressing the issues considered relevant to patients with CL was constructed. Considering the range of manifestations for the different cutaneous *Leishmania* species around the world, we chose to develop a questionnaire focused on the localized form of CL since it is the most common form of the disease in Brazil. The questionnaire items were generated in Brazilian Portuguese, in order to be tested in the native language of the target patients.

### Tool analysis by health professionals *(experts)*

The purpose of this step was to identify if the 27 questionnaire items generated in the previous step were sufficiently comprehensive from the perspective of professionals involved in the treatment of these CL patients. This round of evaluation represents the validation of content, which aims to examine whether all relevant aspects of the disease in focus are adequately represented in the instrument [13]. In other words, one must check if the items in the questionnaire sample the complete range of the attribute under study [14], which is usually judged by a panel of *experts* [13–15]. Thus, a team of five health professionals of the staff of the Leishmaniasis Referral Centre, *René Rachou Institute*, *Fundação Oswaldo Cruz*, directly involved in the care of these patients, consisting of three medical doctors, one nurse and one microbiologist, was invited to evaluate the questionnaire.

This analysis was guided by a semi-structured script and the *experts* were asked to evaluate each item according to an appropriate scale. The relevance of each item was evaluated using an item-level Content Validity Index (I-CVI), an index based on the proportion of items ranked 3 or 4 by *experts* [16] from a scale of 1 to 4 (1 = not relevant, 2 = somewhat relevant, 3 = quite relevant, 4 = highly relevant). Then, a multi-rater *Kappa* statistic (κ) measuring the proportion of *experts* who agreed on that aspect was performed for each item. Each item on the questionnaire was rated as “fair,” “good,” or “excellent,” based on the following rating criteria: fair, κ < 0.59; good, κ = 0.60–0.74; excellent, κ > 0.74 [17]. The items rated as "fair" were deleted. In that way, this process allowed for the removal questions from the questionnaire that were considered not relevant to the disease context. Questions considered unclear or ambiguous by the *experts* were reformulated. Furthermore, suggestions about new items, according to the disease characteristics, were suggested.

### Tool evaluation performed by the patients

After the content validation, which was performed by the health professional team, a face validity process was performed applying a semi-structured interview to patients affected by CL. Ten volunteer patients, consecutively attended at the Leishmaniasis Referral Centre and representing the predominant disease spectrum of CL, were asked about the clarity and relevance of the questionnaire’s items using a guide. Face validity was conducted in a two-step process intended to provide insight into how potential participants might interpret and respond to the items, considering the grammar, syntax and organization of the tool [14]. Initially, the questionnaire resulting from the *expert* analysis was applied individually to five patients, to assess their comprehension of each item that could be considered difficult, unclear, or embarrassing, or that contained difficult words (yes/no questions). For each item, a Concordance Index (c-index) was calculated, and items with a c-index <80% were discarded while items with a c-index between 80% and 99% were reworded [18]. Only items with perfect agreement (c-index = 100%) remained unchanged. Moreover, other relevant items could also be suggested by the patients. This process resulted in a new version of the questionnaire that was tested again with another five patients. In this second round, at the end of each interview, the subjects were asked about their general impressions of the questionnaire and, specifically, about how clear the questions and response choices were. After these two pre-test stages, the questionnaire was considered suitable to be applied in a pilot study.

### Pilot study

The survey was conducted in a leishmaniasis referral center, *Instituto René Rachou*, *Fundação Oswaldo Cruz*, in Belo Horizonte, Minas Gerais, Brazil. The sample size was estimated based on the desired precision of Cronbach’s alpha [19]. Minimum sample size to satisfy the requirement of twenty-seven-item instrument to obtain a 95% confidence interval (CI) for Cronbach’s alpha about 0.90 [20] with a desired CI width of about 0.06 was estimated to be ninety-two individuals. Taking into consideration non-response/attrition, one hundred consecutive patients with ages above 18 years, presenting localized parasitologically confirmed CL and with treatment initiated between five and 90 days before the interview were invited to be included in this pilot study between December 2015 and May 2017. Patients with other non-CL-related wounds and cognitive problems that hindered comprehension of the questionnaire were excluded. The pilot version of the questionnaire was applied to each patient individually in a reserved room in the outpatient referral center by the same trained interviewer.

To answer the questionnaire, the patients were asked to consider all the events occurring since the onset of the symptoms of CL. For each question, five possible answers were always presented: never = 0, almost never = l, sometimes = 2, often = 3, and too often = 4; very good = 0, good = l, reasonable = 2, bad = 3, and too bad = 4; or nothing = 0, slightly = 1, not too much or too little = 2, moderately = 3, and extremely = 4. A final score was generated by summing the numerical response codes of all items. The impact of the disease is directly related to the final score obtained, so that the higher the value, the greater the CL impact. The time taken to answer the questionnaire was computed at this step.

To test the criterion validity, that is, the degree of agreement between the results obtained by the new instrument compared to a pre-existing instrument for impact assessment [13–15], we applied a Visual Analogue Scale (VAS) to measure the general impact of CL on patients' lives. Using a 10-cm horizontal axis where zero indicates the maximum degree of dissatisfaction and 10 is the maximum satisfaction with life, the patients were asked to indicate the number that best represented their perception. The lower the score the greater the impact of the disease.

### Data management and statistics

Initially, descriptive analyses were conducted to characterize the patients involved and the aspects of the disease. The psychometric properties of the instrument were tested using the appropriate statistical methods, as described below.

#### Criterion validity

Refers to the extension in which the punctuation of an instrument relates to the “gold standard” [13].

The Spearman correlation coefficient and the 95% confidence interval (95% CI) for the correlation coefficient were used to compare the two tools (VAS and the questionnaire). A correlation of at least 0.70 was classified as good [13].

#### Internal consistency

Measures how the items of an instrument are correlated (homogeneity), measuring the same construct (only one dimension), or evaluating the underlying constructs (multi-dimensional).

The item-total correlation, which is the correlation of each item with the total score of the instrument without that item, was computed. The existence of items with correlation coefficients less the 0.3 pointed to the possible lack of homogeneity among the questions in the instrument.

Cronbach’s alpha coefficient was calculated in this case; each item was individually excluded from the instrument (alpha if item deleted). Cronbach alpha values higher than 0.5, after the exclusion of the item, were considered as definitive exclusion criteria for this item [21].

To determine the instrument´s dimensionality and to verify how the items are related, the ICLUST algorithm was used; ICLUST is an analytical approach specifically developed for group questionnaire items [22]. To obtain the best structure for the instrument, such as consistent and homogeneous items, hierarchical cluster analysis through ICLUST, using polychoric correlations among the 27 items of the instrument, was performed. From this result, clusters with lower alpha values (the average of all the possible split-half reliabilities of an instrument) and lower beta values (the worst split-half reliability) were deleted, until new algorithms resulted in a structure with alpha coefficient values higher than 0.70 [21] and beta coefficient values higher than 0.50 [23] for the main cluster. The adequacy of the dimensional structure was evaluated by the Cluster fit, Pattern fit and RMSR indexes. Thus, the final structure of the instrument was defined.

#### Construct validity

Is determined by the existence of score relationship of an instrument with other measures derived from theoretical hypotheses about the construct that is being measured [24]. In other words, it aims to verify the degree to which the proposed instrument measures the construct for which it was delineated to measure [25]; in this case, it aims to verify the impact of CL on the patient. It may be evaluated in two ways: through the correlations expected among measures and through the differences in the punctuation among “known groups” [13].

The Spearman correlation coefficient and the 95% confidence interval (95% CI) for the correlation coefficient were used to verify the correlation between the score for each item and the cluster in which this item was allocated. The construct´s validity was evaluated as sufficient when higher correlations were obtained between the item and the cluster where it was allocated, and lesser correlations between the item and the opposite cluster where it was allocated [14].

Another approach used to test the validity of the construct was to compare the scores obtained by the instrument (and respective subscales/clusters) with sociodemographic and clinical variables already acknowledged as indicative of the impact of the disease. The Shapiro-Wilk and Kolmogorov-Smirnov tests were used to verify the normality of these data. Then, analysis of variance using tests for non-parametric variables (Wilcoxon signed-rank test and Kruskal-Wallis) were performed to verify the differences among the mean scores obtained in accordance with each variable. A level of significance of 0.05 was used.

All the analyses were performed with the R program version 3.4.0 (The R Foundation for Statistical Computing http://www.r-project.org/) using the “psych” package. The Graphviz (Graph Visualization Software http://www.graphviz.org/) program version 2.38 was used for the graphic visualization of the diagrams.

## Results

**Fig 1:**
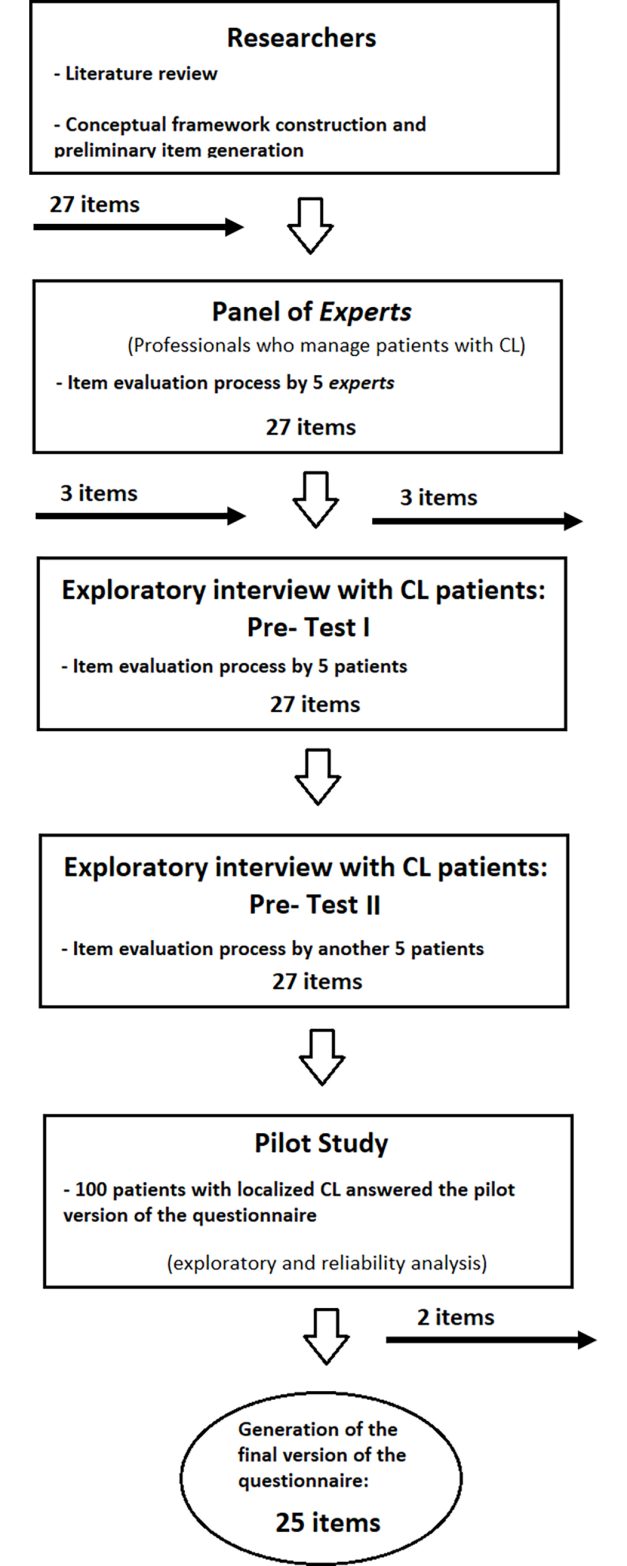
Summary of development of the impact assessment tool for patients with cutaneous leishmaniasis.

Fig 1 shows the search steps in a flowchart.

### Literature review

We identified four studies [1,2,9,10] measuring quality of life and one measuring the psychological impact of cutaneous leishmaniasis [3]. Four different instruments were described in these studies. They were the following: Dermatology Life Quality Index (DLQI) (n = 3), Dermatology Quality of Life Questionnaire (DQL) (n = 1); Body Image Satisfaction Questionnaire (BISS) (n = 1) and Hospital Anxiety Depression Questionnaire (HAD questionnaire) (n = 1). The mains aspects addressed by the instruments were CL symptoms, feelings, daily activities, leisure activities, work or school, personal relationships and treatment.

No instrument included questions related to the economic impact or patient’s satisfaction with health services. Moreover, none of these instruments were developed specifically to evaluate patients with cutaneous leishmaniasis.

Thus, these studies were used to draw an overview of existing instruments and guide the development of the minimum scope of the questionnaire.

### Conceptual framework development and initial questionnaire item generation

The most relevant themes to assess the impact of cutaneous leishmaniasis were hypothesized based on the literature review and the inputs provided by health professionals and patients as follows: global (G), physical and functional limitations (PF), occupational (O), emotional (E), economic (Ec), social (S), impact of treatment (IT) and satisfaction/assessment with health services (SHS). The items were created considering each of these concepts related to the impact of localized cutaneous leishmaniasis for patients. An initial 27-item questionnaire was proposed, and the questions were grouped into the following categories: G (3 items), PF (5 items), O (3 items), E (3 items), Ec (3 items), S (3 items), IT (2 items) and SHS (5 items).

### Tool analysis by health professionals *(experts)*

The evaluation performed by the panel of *experts* and the content validity index obtained are shown in Table 1.

**Table 1:** Content validity index based on *experts* evaluation of the initial items.

Item	Rating by *experts*	Number rating of 3 or 4	I-CVI	Pc	K*	Evaluation
	A B C D E					
**G1**	4 4 4 4 4	5	1.00	0.031	1.00	excellent
**G2**	4 4 3 4 3	5	1.00	0.031	1.00	excellent
**G3**	4 4 3 3 4	5	1.00	0.031	1.00	excellent
**PF4**	4 4 3 4 4	5	1.00	0.031	1.00	excellent
**PF5**	4 4 4 4 4	5	1.00	0.031	1.00	excellent
**PF6**	3 4 2 3 2	3	0.60	0.313	0.42	fair
**PF7**	3 3 2 4 4	4	0.80	0.156	0.76	excellent
**PF8**	4 4 4 4 4	5	1.00	0.031	1.00	excellent
**E9**	4 4 4 4 4	5	1.00	0.031	1.00	excellent
**E10**	4 4 3 4 4	5	1.00	0.031	1.00	excellent
**E11**	3 4 3 4 4	5	1.00	0.031	1.00	excellent
**O12**	4 4 4 4 4	5	1.00	0.031	1.00	excellent
**O13**	3 4 4 4 4	5	1.00	0.031	1.00	excellent
**O14**	2 2 2 2 2	0	0.00	0.031	-0.03	fair
**Ec15**	4 4 3 3 4	5	1.00	0.031	1.00	excellent
**Ec16**	4 4 2 4 2	3	0.60	0.313	0.42	fair
**Ec17**	4 4 2 3 4	4	0.80	0.156	0.76	excellent
**S18**	2 4 1 4 2	2	0.40	0.313	0.13	fair
**S19**	4 4 3 4 4	5	1.00	0.031	1.00	excellent
**S20**	4 4 3 4 4	5	1.00	0.031	1.00	excellent
**IT21**	3 4 4 4 4	5	1.00	0.031	1.00	excellent
**IT22**	4 4 3 4 4	5	1.00	0.031	1.00	excellent
**SHS23**	3 4 1 4 4	4	0.80	0.156	0.76	excellent
**SHS24**	4 4 3 4 4	5	1.00	0.031	1.00	excellent
**SHS25**	2 2 1 4 2	1	0.20	0.156	0.05	fair
**SHS26**	3 4 3 4 4	5	1.00	0.031	1.00	excellent
**SHS27**	4 4 2 4 4	4	0.80	0.156	0.76	excellent

Note: Letters A to E represent individual *experts*; 1 = not relevant, 2 = somewhat relevant, 3 = quite relevant, and 4 = highly relevant; CVI, content validity index; I-CVI, Item-level CVI; Pc, probability of chance agreement; K*, the multi-rater kappa statistic; G, global domain; PF, physical and functional domain; E, emotional domain; O, occupational domain; Ec, economic domain; S, social domain; IT, impact of treatment domain; SHS, satisfaction/assessment with health services domain.

On the basis of the CVI results, five items (PF6, O14, Ec16, S18 and SHS25) were excluded and the following four items were added: *“Did you have to change the style of dressing because of other people's prejudices about their skin wounds*?*”*; *“Have you ever felt guilty or insecure about cutaneous leishmaniasis*?*”*, *“How much do you care about the need to seek health services for the treatment of cutaneous leishmaniasis*?*”* and *“Do you consider that Cutaneous Leishmaniasis has financially damaged you family´s budget*?*”* The item “*What did you think about the way it was hosted by the health services in search of the diagnosis and treatment of cutaneous leishmaniasis*?*"* was divided into two items, considering the diagnosis and treatment separately. Finally, some items were reformulated to reduce ambiguity (G2, G3, PF7, EC15, IT21 and SHS24).

### Tool evaluation performed by the patients

The ten patients presenting cutaneous leishmaniasis in the interviews had different educational levels. In the first round with five patients, and based on the Concordance Index, no items were deleted, suggesting good item acceptability. Four items were considered unclear (G2, PF6, IT20 and SHS25), while the SHS25 item was considered difficult to understand and containing unknown words. These items were reworded based on the comments of the patients. One item was mentioned by patients as a cause of shame or discomfort (PF7), but it was not excluded because of its relevance. Overall, the patients expressed positive comments regarding the questionnaire and no new item was suggested. During the second round, with five other patients, the Concordance Index was considered perfect. S1 File shows the set of items initially proposed and the changes performed according to the expert and patient inputs.

### Pilot study

One hundred patients with localized CL answered the pilot version of the questionnaire. The mean duration of the interview was seven minutes (ranging from two to 20 minutes), and no difficulty was perceived by the patients related to age or intellectual ability. The sample was dominated by male (71%) and 50% of the patients were until 42 years old. About education level, and 54% of the patients had the corresponding to primary school or lower. Regarding to disease severity, most patients had an ulcerative (83%) and single lesion (69%). Furthermore, all patients were treated with meglumine antimoniate: 52% of them with the intralesional infiltration approach and the other 48% using the intravenous route. The demographic and clinical characteristics of the patients are detailed in Table 2.

**Table 2:** Demographic characteristics of the patients included in the psychometric validation study.

Characteristics	N = 100
**Age**	
18 to 30	26
31 to 42	25
43 to 58	26
59 to 81	23
**Gender**	
Female	29
Male	71
**Marital status**	
Single	28
Married	57
Divorced	12
**Highest education level completed**	
Primary school or lower	54
High school	27
College or higher	19
**Work status**	
Employed	76
Unemployed	2
Retired	14
Student	3
Housewife	4
**Disease severity**	
**Number of lesions**	
One lesion	69
Two lesions	14
Three or more lesions	17
**Lesion appearance**	
Ulcerative	83
Non-ulcerative	17
**Presence of secondary infection**	9
**Relapse after cure**	4
Therapy	
Meglumine antimoniate Intralesional	52
Meglumine antimoniate Intravenous	48

### Criterion validity

After comparing the score obtained with the questionnaire and the VAS, a linear correlation was observed (r = 0.82), suggesting that the two instruments produce similar results.

### Internal consistency

Table 3 presents the results of item-total correlation, alpha-if-item-deleted analysis, and other item-level analyses.

**Table 3:** Item-level analysis of questionnaire in development.

Item	Frequency of response by category n (%)					Range	Median	Alpha if item deleted	Correlation Item-total	Correlation Item-VAS
	0	1	2	3	4					
**G1**	26(26)	47(47)	22(22)	4(4)	1(1)	0–4	1	0.85	0.43	0.30*
**G2**	31(31)	16(16)	1(1)	26(26)	26(26)	0–4	3	0.84	0.64	0.68*
**G3**	50(50)	43(43)	5(5)	2(2)	0(0)	0–3	0.5	0.85	0.30	0.12
**PF4**	65(65)	4(4)	14(14)	8(8)	9(9)	0–4	0.0	0.85	0.55	0.47*
**PF5**	11(11)	10(10)	34(34)	21(21)	24(24)	0–4	2.0	0.85	0.47	0.41*
**PF6**	54(54)	6(6)	5(5)	15(15)	20(20)	0–4	0.0	0.84	0.63	0.52*
**PF7**	76(76)	1(1)	15(15)	2(2)	6(6)	0–4	0.0	0.85	0.35	0.32*
**E8**	52(52)	5(5)	27(27)	9(9)	7(7)	0–4	0.0	0.85	0.38	0.33*
**E9**	23(23)	12(12)	31(31)	14(14)	20(20)	0–4	2.0	0.85	0.56	0.46*
**E10**	76(76)	10(10)	3(3)	5(5)	6(6)	0–4	0.0	0.85	0.49	0.35*
**E11**	69(69)	5(5)	17(17)	5(5)	4(4)	0–4	0.0	0.85	0.50	0.26*
**O12**	46(46)	7(7)	13(13)	11(11)	23(23)	0–4	1.0	0.85	0.36	0.32*
**O13**	49(49)	7(7)	4(4)	14(14)	26(26)	0–4	1.0	0.85	0.56	0.54*
**Ec14**	27(27)	24(24)	9(9)	20(20)	20(20)	0–4	1.0	0.85	0.54	0.51*
**Ec15**	68(68)	10(10)	4(4)	9(9)	9(9)	0–4	1.0	0.85	0.52	0.52*
**Ec16**	86(86)	0(0)	2(2)	5(5)	7(7)	0–4	0.0	0.85	0.35	030*
**S17**	73(73)	0(0)	9(9)	6(6)	12(12)	0–4	0.0	0.85	0.34	0.33*
**S18**	65(65)	2(2)	15(15)	9(9)	9(9)	0–4	0.0	0.84	0.60	0.48*
**S19**	84(84)	9(9)	4(4)	2(2)	1(1)	0–4	0.0	0.85	0.45	0.33*
**IT20**	50(51)	35(35.7)	10(10.2)	1(1)	2(2)	0–4	0.0	0.86	0.04	0.12
**IT21**	53(53)	15(15)	18(18)	6(6)	8(8)	0–4	0.0	0.85	0.45	0.43*
**IT22**	53(53)	12(12)	7(7)	13(13)	15(15)	0–4	0.0	0.85	0.48	0.51*
**SHS23**	65(65)	27(27)	3(3)	4(4)	1(1)	0–4	0.0	0.85	0.26	0.18
**SHS24**	76(76)	17(17)	3(3)	3(3)	1(1)	0–4	0.0	0.86	0.09	0.18
**SHS25**	47(47)	15(15)	11(11)	18(18)	9(9)	0–4	1.0	0.86	0.25	0.20*
**SHS26**	55(55)	5(5)	8(8)	5(5)	27(27)	0–4	0.0	0.86	0.25	0.32*
**SHS27**	57(57)	4(4)	6(6)	6(6)	27(27)	0–4	0.0	0.87	-0.09	0.04

*p< 0.05

The item-total correlation ranged from -0.09 to 0.64. Although some item-total correlations were strong, six of 27 items had item-total correlations below 0.3, suggesting little relation to a possible general factor.

**Fig 2:**
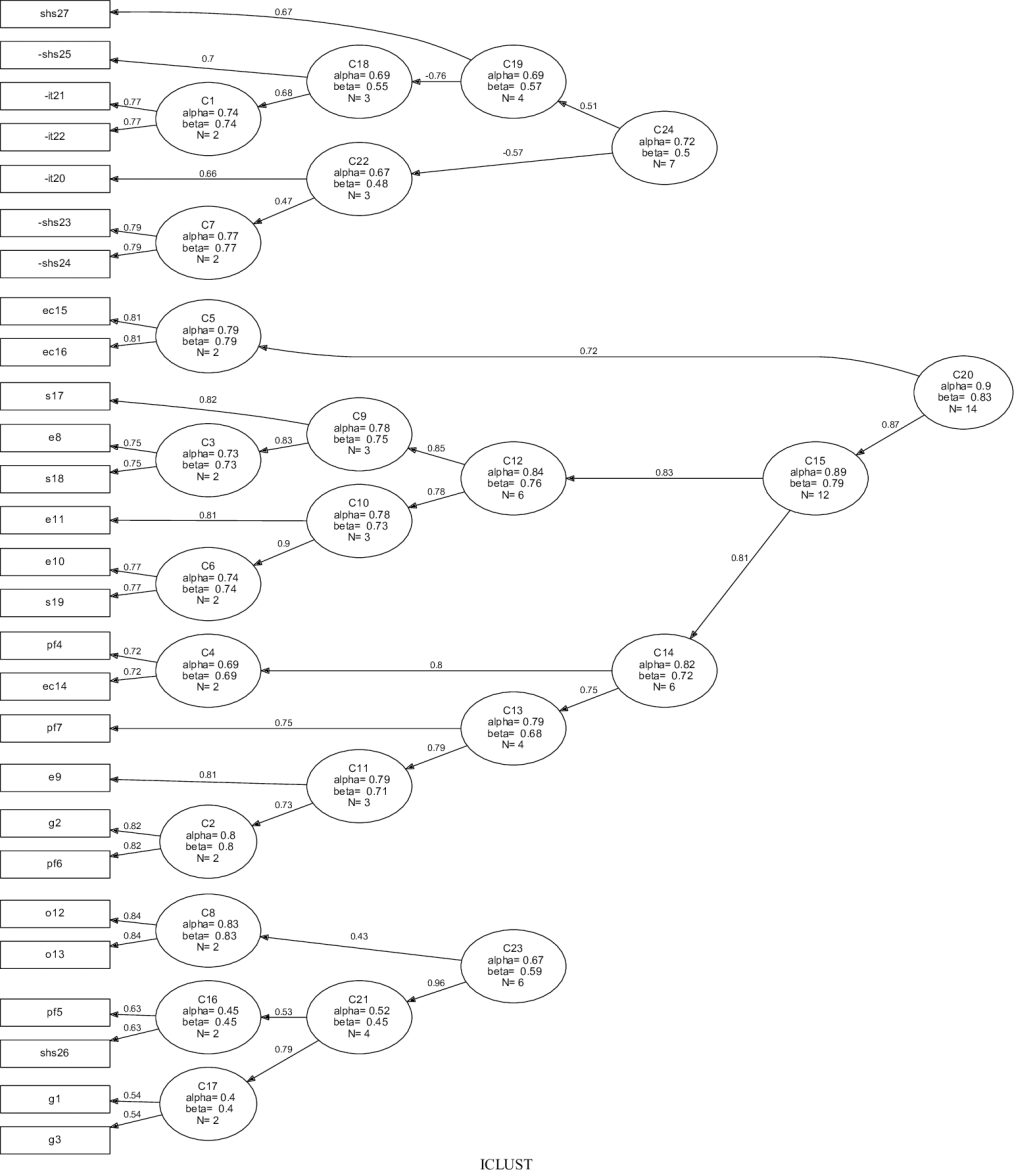
Questionnaire item clustering (n = 100). Clusters were formed using the ICLUST algorithm, and three higher-order clusters were identified: C23 = Bad theoretical meaning; C24 = Satisfaction with health services and treatment; C20 = Social, economic, functional and emotional impacts of the disease.

Based on cluster analysis, Fig 2 illustrates how the items are related to another item. Initially, three clusters were formed (C23, C24 and C20), with their eigenvalues equal to 2.6, 2.8 and 6.6, respectively.

**Fig 3:**
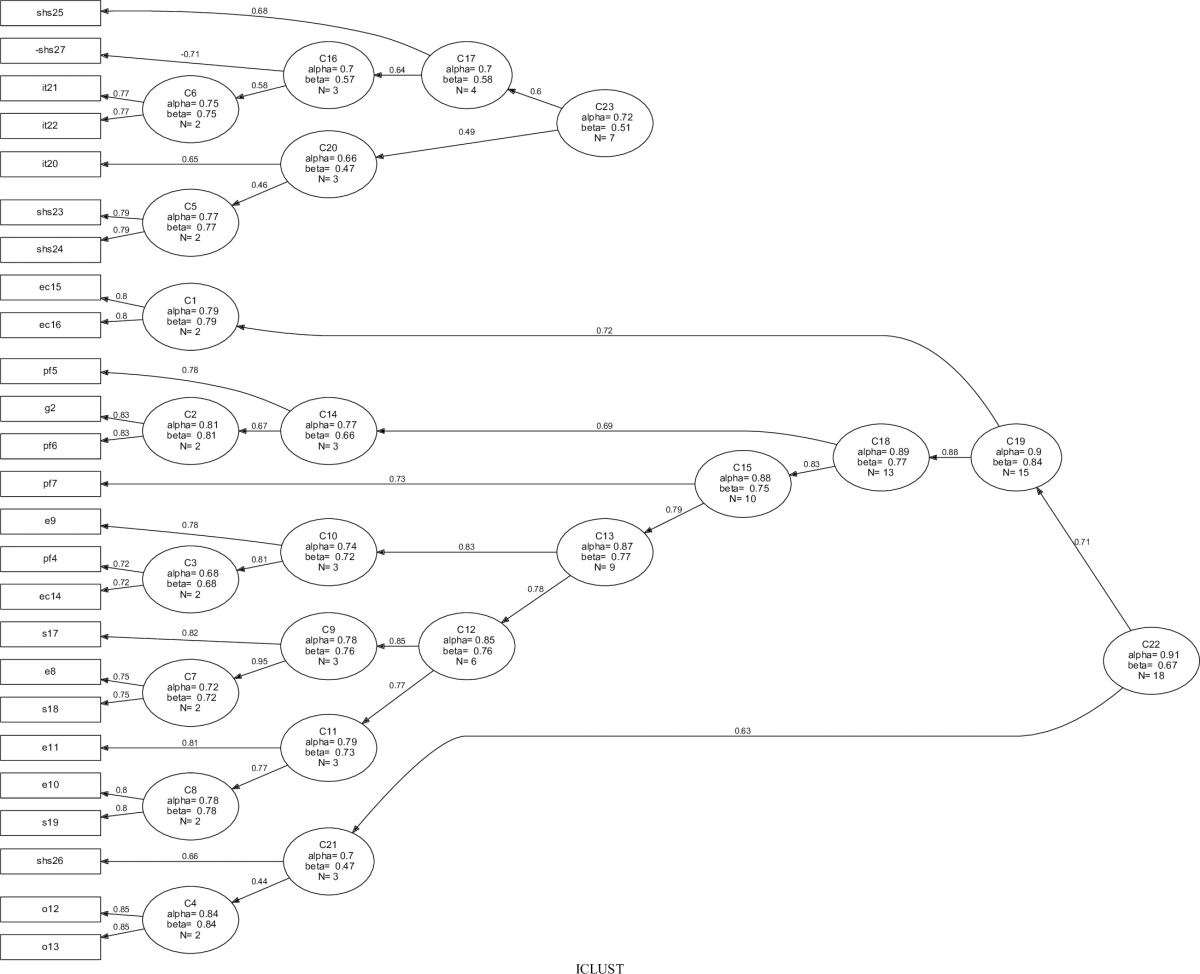
Questionnaire item clustering (n = 100). Clusters were formed using the ICLUST algorithm, after deleting cluster C17 (items G1 and G3), resulting in two higher-order clusters: C23 = Perception about health services and treatment; C22 = General impacts.

However, the C23 cluster in this analysis presented low internal consistency (Cronbach alpha 0.67); in addition, the items in this cluster do not contribute to a well-defined theoretical model. Proceeding with the criterion defined in the methods section, the C17 cluster (formed by the G1 and G3 items) was deleted because it showed lower alpha and beta values. Later, a new ICLUST algorithm was generated and Fig 3 illustrates this new result.

After a new regrouping of the items, two main clusters emerged (C22 and C23, with autovalues of 7.2 and 2.9, respectively), which were consistent (alpha > 0.70), homogeneous (beta > 0.50) and contributing to a well-defined theoretical model. The C22 cluster gathered items related to the general impact of CL and the C23 cluster gathered items related to the perception of the patients about the treatment and health services. The item SHS27 was allocated at the C23 cluster, but it is related to this cluster in an inverse form. Lastly, Cronbach´s alpha coefficient for the questionnaire, as a whole, was 0.86.

### Construct validity

When evaluating the correlation of each item with the cluster where it was allocated and with the underlying cluster, it was possible to verify that each item was strongly correlated with its own cluster. Table 4 shows that the general impact scale score for CL was significantly different among patients reporting adverse effects during CL treatment and those reporting work losses due to the disease itself. Additionally, the perception scale about the health and treatment services discriminated individuals receiving different treatments (antimoniate meglumine intravenously or through intralesional infiltration), patients who reported adverse effects related to the medication, people who had to purchase medications through the course of the disease and people who had to spend money on transportation for the appointments and CL treatments.

**Table 4:** Mean comparisons of the questionnaire and sub-questionnaire (scales) scores against categorical criterion-related validity measures.

Variables			Questionnaire	
		CL general impacts	Perception about health services and treatment	Total Score
**Therapy**		p = 0.487*	p = 0.002*	p = 0.253*
**Adverse events**		p = 0.036*	p< 0.001*	p = 0.001*
**Health insurance**		p = 0.289*	p = 0.662*	p = 0.451*
**Missed work related to CL**		p< 0.001*	p = 0.314*	p = 0.001*
**Gender**		p = 0.211*	p = 0.208*	p = 0.113*
**Number of lesions**		p = 0.949**	p = 0.566**	p = 0.938**
**Need to spend with medical consultation**		p = 0.076*	p = 0.076*	p = 0.054*
**Need to spend with medical exams**		p = 0.715*	p = 0.079*	p = 0.401*
**Need to spend with drug therapy**		p = 0.083*	p< 0.001*	p = 0.045*
**Need to spend on transportation related to treatment of CL**		p = 0.352*	p = 0.029*	p = 0.172*

* Mann Whitney

** Kruskal-Wallis

Based on the results of pilot study, only one item had missing values (item 20) which represented 0.07% of the total values of data. Missing data were handled using mean imputation. In general, the proposed questionnaire (S2 File), now called the Cutaneous Leishmaniasis Impact Questionnaire (CLIQ), showed good internal consistency and reliability.

## Discussion

CL is not a lethal disease, but, similar to other tropical neglected diseases, it might cause high morbidity, especially related to psychosocial health, because of its association with stigmatization and discrimination, especially in places with reduced access to health services and social vulnerability [26]. The completion of this study was motivated by the gaps in the present methods used to evaluate the burden of CL, which only consider the resulting physical disabilities [4, 8, 27, 28]. An important consideration from the literature is that the DALY estimates (Disability-adjusted life year) do not consider the social stigmatization or the emotional and financial impacts of CL [8, 26, 28]. Therefore, it was recommended to develop an instrument that allows a broad and standardized evaluation of the impact of CL, guarantying data comparability among the endemic countries and strengthening future conclusions [4].

Many instruments have been validated to evaluate the impact of dermatologic diseases on quality of life (DLQI [12], DQL [29], Skindex-29 [30], PLSI [31], NailQol [32], Scalpdex [33], VLU-Qol [34], and FLQA-d [35]). However, to our knowledge, none of them have been specifically designed for CL. Seeking mainly to include the relevant CL aspects not covered by the generic instruments, we describe the development and the first validation of a new questionnaire focused on CL, the Cutaneous Leishmaniasis Impact Questionnaire. In this context, knowing the disease well and being able to count on the help of *experts* and patients for the formulation of this questionnaire was fundamental. In the end, both the clinical and contextual relevance of the items, evaluated qualitatively, and the statistical results were taken under consideration in the decisions to maintain or remove items, contributing to the obtained questionnaire.

The process of constructing a questionnaire that is used in the social sciences frequently involves a deep analysis of a set of items, and those with recognized relevance will remain in the instrument. This criterion might be rational, empiric and factorial [36]. The best statistical methods to verify the structure and dimensionality of the data collection instruments still are a great gap in the scientific literature [37, 38]. There are many solutions for this problem, but none of them produce a result that is 100% trustworthy [39]. Some examples of techniques to evaluate the dimensionality of questionnaires are factor analysis, especially component analysis, and cluster analysis. The factorial analysis aims to identify latent variables that subjugate the scales, in other words, use correlation or covariance matrices to identify constructs that explain the data observed [40]. The main components analysis is frequently interpreted in similar terms as the factorial analysis since both estimate factors from the correlation matrix of measured variables to extract components that account for the maximum possible variance in the observed variables [40]. That way, the items with high impacts in a certain factor are combined at the same scale. Additionally, the analysis of the cluster is used to group variables that are perceived in similar ways, forming a more homogeneous set of items in each cluster [41]. This approach combines the two most similar variables, starting from the correlation matrix that was generated, and then calculates the similarity of this composite variable with the remaining variables [42]. Among these, the most conventional method reported by literature is the factorial analysis. However, the items included in this study, are not completely related to psychological traces, which demanded the initial use of the cluster analysis [43]. In this case, we opted to use the ICLUST algorithm, which was specifically developed to join items of questionnaires and was available as a free psych package in statistical software [44]. A great advantage of ICLUST is that the items are only added to a cluster if they increase its internal consistency and factorial homogeneity [45]; in addition, it provides information in diagrams that are easy to interpret [39]. This is a descriptive analysis technique, which is used especially in exploratory model analyses [23] and is aligned with the objective of this phase of this study.

The ICLUST algorithm uses a different strategy to build scales; the criterion is the time until the alpha-beta rules cease to be met, at which point a cluster is finished [46]. However, a problem originating from an inability to calculate the beta and to conduct a factorial analysis, a priori, is that alpha can be high even if the components are not correlated, that is, when there is no general factor [47].

Through visual inspection of the diagram generated by the ICLUST, we verified that some variables that were initially organized by the researchers in distinct categories were grouped together, indicating that the patients possibly experienced them in a similar way. It is interesting to note that both items identified by the cluster analysis with low consistency and homogeneity were not directly related to the impact of CL but instead to the perception of general health and life satisfaction, independent of disease. Thus, after eliminating the items that were allocated in a cluster with low alpha and beta values, we obtained an acceptable internal consistency (alpha >0.70) [21] and a better item performance. Mathematically, Cronbach´s alpha varies from 0 to 1, and it is an adjusted proportion of the total variance of the item´s scores [48]. Contrary to the recommended value for alpha, which is well established by the literature, the ideal value for the beta coefficient is still poorly explored. Revelle (1979) suggests that a lower value for the beta coefficient might be less than 0.50, which represents less than 50% of the variable scale associated with the first reference factor. In addition, it is suggested that the difference between the alpha and the beta values must be less than 0.10 to assert the unidimentionality of a questionnaire [47]. Differences between alpha and beta that are higher than 0.15 and 0.20, and beta values lower than 0.50, point to the presence of more than one scale, or underlying construct, in the same questionnaire [46]. Therefore, the Cutaneous Leishmaniasis Impact Questionnaire presents two reliable and homogeneous scales, one evaluating the general impact of the disease and another evaluating the perception about the treatment and health services. To observe the proximity between the variables in a graphic form clarifies how they are related from the patient´s perspective.

As we developed the questionnaire, we aimed to obtain a numeric sum, which represents the impact of localized cutaneous leishmaniasis perceived by the patients, allowing a comparison of the data from the health services in different areas, at different points in time and among patients receiving different treatments. Thus, the 25 items, varying on a scale from 0 to 4, will produce a score from 0 to 100 points, with 0.86 reliability. When presenting two underlying constructs, both scales´ scores should also be evaluated separately.

Some questions are known to be important for comprehending the impact of CL, such as the toxicity related to the treatment, the high cost of the medications and the difficulty confirming the diagnosis, which can delay starting the correct treatment [49]. In addition, issues related to the lack of material, technical or infrastructure resources by the public health services for CL treatment should also be considered in the disease impact assessment [11]. In this sense, our questionnaire, by evaluating the perception of the health and treatment services, captured these matters well. First, it is worth noting that the mean score was significantly different among patients treated with meglumine antimoniate administered intravenously in comparison with patients treated with intralesional infiltration, suggesting that the impact of the disease is different according to the type of treatment. Additionally, both the total score from the questionnaire and the score per scale were different between groups that reported or denied the occurrence of adverse effects related to the medication, confirming that the toxicity of the medication negatively impacts the lives of the patients. Patients who had extra expenses with the disease due to the purchase of medication had higher scores in the questionnaire and on the scale of the perception of health and treatment services, which shows the importance of this aspect from the patient´s perspective. The need to pay for the doctor´s appointments, despite showing borderline statistical significance, reflects the tendency that this variable can also impact the lives of people with CL. Lastly, the general impact scale score for CL was higher for those patients who reported a loss of work days, suggesting that professional damage contributes to the impact of CL.

Regarding the instrument´s format, considering the present developmental phase of the questionnaire, which includes many successive steps from the suggested modifications by the stakeholders, it was important to apply it in the form of an interview about this aspect, which could represent a limitation for the dissemination of its use or even a source of bias, it is important to emphasize that all the interviews were completed by the same researcher in an attempt to reduce artifacts related to biased information. Furthermore, new studies should be conducted with auto-application of the questionnaire, which could generate additional information about the usefulness of the instrument. Another possible limitation of this study was the impossibility of conducting test-retest assessment; for instance, the course of treatment could cause fluctuations in the responses and the consistency could be lowered. In addition, our data were obtained at a reference center for the treatment of CL, which is a place with all the infrastructure needed to diagnose and treat the condition being studied. To apply this instrument in other settings and to evaluate the patient´s perception of other primary attention health care services could provide additional information for planning of health policies to promote comprehensive access. Lastly, the main limitation of this research is that the developed scale was not compared to any other instrument; however, we believe that evaluating the correlation of the studies with the VAS provides an initial basis to discuss criterion validity. Comparing this questionnaire with the DLQI in future studies would clarify the specificity of the Cutaneous Leishmaniasis Impact Questionnaire.

From the evidence presented here, we consider that the Cutaneous Leishmaniasis Impact Questionnaire has adequate validity and good internal consistency, representing an important step towards the development of an instrument aimed at the quantification of life impact generated by leishmaniasis. As we admit that the psychometric analyses were tested and modified from the contributions of relatively small samples of patients, which still need new studies to replicate and confirm the results, we are cautious to characterize it as an initial study. Thus, additional research needs to be done for the complete validation of the questionnaire. Future studies involving a higher sample size and different cultural contexts are also important to confirm the strength of the instrument and eventually to generate modifications and additions.

In the clinical trials context, the use of instruments such as this, allows the researcher to evaluate the impact generated by different types of treatment at a certain moment, or in different moments [50], or even in different populations. Lastly, the incorporation of the patient´s perspective in the study design, and ultimately in the formulation of the clinical management recommendations, is needed to achieve legitimate health policies that attend to the needs of society. Even more, this is about a strategy needed to guarantee policies that incorporate the local context, in relation to the broad spectrum of patients, diseases and levels of complexity of the health system [50].

## Conclusion

The Cutaneous Leishmaniasis Impact Questionnaire has been developed with extensive patient and expert input and demonstrates evidence of initial validity and reliability.

## Supporting information

S1 File Bank of items initially proposed by the researchers and proposed final structure after the evaluation of the items by *experts* and patients. (PDF)

S2 File Cutaneous Leishmaniasis Impact Questionnaire (CLIQ). (PDF)
