# Supplementary material for: Development and initial validation of a cutaneous leishmaniasis impact questionnaire
Source: PLoS One. 2018 Aug 30;13(8):e0203378. doi: 10.1371/journal.pone.0203378 (PMC6117079; doi:10.1371/journal.pone.0203378)
Supplement: S1 File — (PDF) [file pone.0203378.s001.pdf]

S1 File. Bank of items initially proposed by the researchers and proposed final structure after the evaluation of the items by *experts* and patients.

Categories	Bank ofinitial itens	Situation	Final structure proposed after qualitative steps
Global	**G1-Como você avalia a sua condição de saúde atual?**<br>**How do you evaluate your current health condition?*	Item not changed	**G1-Como você avalia a sua condição de saúde atual?**<br>**How do you evaluate your current health condition?*
	**G2-A sua condição de saúde prejudica o seu bem-estar geral?**<br>**Does your health condition affect your overall well-being?*	Reformulated item	**G2- A Leishmaniose cutânea prejudicou o seu bem-estar geral?**<br>**Has Cutaneous Leishmaniasis affected your overall well-being?*
	**G3-Como você avalia sua própria vida em termos de felicidade, realização pessoal e satisfação com a vida?**<br>** How do you evaluate your own life in terms of happiness, personal fulfillment, and life satisfaction?*	Reformulated item	**G3-Como você avalia a sua satisfação com a vida?**<br>** How do you rate your satisfaction with life?*
Physical / Functional	**PF4-Você já teve dificuldade para andar, trocar de roupa ou tomar banho, por causa da (s) ferida (s) na pele?**<br>**Have you had difficulty walking, changing clothes or bathing because of the wound (s) on your skin?*	Item not changed	**PF4-Você já teve dificuldade para andar, trocar de roupa ou tomar banho, por causa da (s) ferida (s) na pele?**<br>**Have you had difficulty walking, changing clothes or bathing because of the wound (s) on your skin?*
	**PF5-Você já sentiu dor, ardor, coceira ou incômodo no local da (s) ferida (s) da pele?**<br>**Have you felt pain, burning, itching or discomfort at the site of the skin wound (s)?*	Item not changed	**PF5-Você já sentiu dor, ardor, coceira ou incômodo no local da (s) ferida (s) da pele?**<br>**Have you felt pain, burning, itching or discomfort at the site of the skin wound (s)?*
	**PF6-Com que frequência você procura os serviços de saúde por causa da leishmaniose cutânea?**<br>**How often do you seek health services because of Cutaneous Leishmaniasis?*	Item excluded	
	**PF7- Com que freqüência a leishmaniose cutânea atrapalhou a sua prática de atividade física?**<br>**How often has Cutaneous Leishmaniasis interfered with your physical activities?*	Reformulated item<br>Allocated in PF6	**PF6- A leishmaniose cutânea interferiu na sua prática de atividade física?**<br>**Has Cutaneous Leishmaniasis interfered with your physical activities?*
	**PF8-Você já teve dificuldade na relação sexual por causa da (s) ferida (s) na pele?**<br>**Have you ever had difficulty during sexual intercourse because of the skin wound(s)?*	Item not changed<br>Allocated in PF7	**PF7-Você já teve dificuldade na relação sexual por causa da (s) ferida (s) na pele?**<br>**Have you ever had difficulty during sexual intercourse because of the skin wound (s)?*
Emotional	**E9-Você já se sentiu envergonhado por causa da (s) ferida (s) na pele?**<br>**Have you ever felt embarrassed because of the skin wound (s)?*	Item not changed<br>Allocated in E8	**E8-Você já se sentiu envergonhado por causa da (s) ferida (s) na pele?**<br>**Have you ever felt embarrassed because of the skin wound (s)?*
	**E10-Você já ficou nervoso, triste ou com medo por causa da leishmaniose cutânea?**<br>**Have you ever felt nervous, sad or scared because of Cutaneous Leishmaniasis?*	Item not changed<br>Allocated in E9.	**E9-Você já ficou nervoso, triste ou com medo por causa da leishmaniose cutânea?**<br>**Have you ever felt nervous, sad or scared because of Cutaneous Leishmaniasis?*
	**E11-Você já sofreu achando que sua aparência é diferente das pessoas que não têm feridas na pele?**<br>**Have you suffered thinking that your appearance is different from people who don´t have wounds on their skin?*	Item not changed<br>Allocated in E10	**E10-Você já sofreu achando que sua aparência é diferente das pessoas que não têm feridas na pele?**<br>**Have you suffered thinking that your appearance is different from people who don´t have wounds on their skin?*
		Suggested item for inclusion	**E11-Você já teve sentimento de culpa ou insegurança por causa da leishmaniose cutânea?**<br>**Have you ever felt guilty or insecure about Cutaneous Leishmaniasis?*
Occupational	**O12-Você já faltou ao trabalho (ou à escola) por causa da leishmaniose cutânea?**<br>**Have you ever missed work (or school) because of Cutaneous Leishmaniasis?*	Item not changed	**O12-Você já faltou ao trabalho (ou à escola) por causa da leishmaniose cutânea?**<br>**Have you ever missed work (or school) because of Cutaneous Leishmaniasis?*
	**O13- A leishmaniose cutânea afetou de alguma forma a sua capacidade de trabalhar (ou estudar)?**<br>**Has Cutaneous Leishmaniasis somehow affected your ability to work (or study)?*	Item not changed	**O13- A leishmaniose cutânea afetou de alguma forma a sua capacidade de trabalhar (ou estudar)?**<br>**Has Cutaneous Leishmaniasis somehow affected your ability to work (or study)?*
	**O14- Com qual freqüência você precisou internar por complicações da leishmaniose cutânea?**<br>**How often have you had to be hospitalized for complication of the Cutaneous Leishmaniasis?*	Item excluded	
Economic	**Ec15- Com qual frequência a leishmaniose cutânea aumentou suas despesas com sua saúde?**<br>**How often has Cutaneous Leishmaniasis increased your health expenses?*	Reformulated item.<br>Allocated in Ec14	**Ec14- A leishmaniose cutânea aumentou de alguma forma seus gastos com sua saúde?**<br>**Has Cutaneous Leishmaniasis somehow increased your health expenses?*
	**Ec16- Você considera que a leishmaniose cutânea impactou negativamente o orçamento da sua família?**<br>**Do you consider that Cutaneous Leishmaniasis has negatively impacted you family´s budget?*	Item excluded<br>Suggested item for inclusion	**Ec15- Você considera que a leishmaniose cutânea prejudicou financeiramente a sua família?**<br>**Do you consider that Cutaneous Leishmaniasis has financially damaged your family´s budget?*

	**Ec17-Com que freqüência você precisou pagar alguém para te substituir em atividades do trabalho ou de casa para ir ao serviço de saúde?** **How often have you had to pay someone to replace you at work or home activities so you could go get health service?*	Item not changed Allocated in Ec16	**Ec16-Com que freqüência você precisou pagar alguém para te substituir em atividades do trabalho ou de casa para ir ao serviço de saúde?** **How often have you had to pay someone to replace you in work or home activities so you could go get health service?*
Social	**S18- Com qual freqüência você tem precisado de suporte da família e amigos desde que teve a leishmaniose cutânea?** **How often have you needed support from your family and friends since you got Cutaneous Leishmaniasis?*	Item excluded	
		Suggested item for inclusion	**S17-Você precisou mudar o estilo de se vestir por causa de preconceito de outras pessoas em relação às suas feridas na pele?** **Did you have to change the style of dressing because of other people's prejudices about their skin wounds?*
	**S19- Com qual freqüência você está evitando atividades sociais com grupos de pessoas por causa da leishmaniose cutânea?** **How often have you avoided social activities with groups of people because of Cutaneous Leishmaniasis?*	Item not changed Allocated in S18	**S18- Com qual freqüência você está evitando atividades sociais com grupos de pessoas por causa da leishmaniose cutânea?** **How often have you avoided social activities with groups of people because of Cutaneous Leishmaniasis?*
	**S20- Você está se sentindo isolado das outras pessoas desde que teve a leishmaniose cutânea?** **Do you feel isolated by others since you got Cutaneous Leishmaniasis?*	Item not changed Allocated in S19	**S19- Você está se sentindo isolado das outras pessoas desde que teve a leishmaniose cutânea?** **Do you feel isolated from others since you got Cutaneous Leishmaniasis?*
Impactof Treatment	**IT21-Como você avalia a medicação que usou no tratamento para leishmaniose cutânea?** **How do you evaluate the medication you used to treat Cutaneous Leishmaniasis?*	Reformulated Item Allocated in IT20	**IT20- O que você acha da medicação usada para tratar a leishmaniose cutânea?** **What do you think about the medication you used to treat Cutaneous Leishmaniasis?*
	**IT22- Com qual freqüência você já passou mal por causa dos remédios que usou para tratar a leishmaiose cutânea?** **How often have you felt sick because of the medications you took to treat Cutaneous Leishmaniasis?*	Item not changed Allocated in IT21	**IT21- Com qual freqüência você já passou mal por causa dos remédios que usou para tratar a leishmaniose cutânea?** **How often have you felt sick because of the medications you took to treat Cutaneous Leishmaniasis?*
		Suggested item for inclusion	**IT22- O quanto te incomoda a necessidade de procurar os serviços de saúde para o tratamento da leishmaniose cutânea?** **How much do you care about the need to seek health services for the treatment of cutaneous leishmaniasis?*
Satisfaction with Health Services	**SHS 23- O que você achou sobre a forma como foi acolhido pelos serviços de saúde em busca do diagnóstico e tratamento da leishmaniose cutânea?** **What did you think about how you were welcomed by the health services when you were seeking diagnosis and treatment of Cutaneous Leishmaniasis?*	Item dismembered in two (SHS23 e SHS24)	**SHS23- O que você achou sobre a forma como foi acolhido pelos serviços de saúde em busca do diagnóstico da leishmaniose cutânea?** **What did you think about how you were welcomed by the health services when you were seeking diagnosis of Cutaneous Leishmaniasis?*
			**SHS24- O que você achou sobre a forma como foi acolhido pelos serviços de saúde em busca do tratamento da leishmaniose cutânea?** **What did you think about how you were welcomed by the health services when you were seeking treatment for Cutaneous Leishmaniasis?*
	**SHS24-Houve demora em conseguir realizar exames, consultas ou internação para o tratamento da leishmaniose cutânea?** **Was there any delay in getting the tests done, medical appointments or hospitalizations for the treatment of Cutaneous Leishmaniasis?*	Reformulated Item Allocated inSHS25	**SHS25- O quanto demorou até realizar exames, consultas ou internação relacionados à leishmaniose cutânea?** **How long has it taken to get the tests done, medical appointments or hospitalizations related to Cutaneous Leishmaniasis?*
	**SHS25-Você teve (ou ainda tem) dificuldade de chegar às unidades de saúde para tratamento da leishmaniose cutânea?** **Have you had (or still have) difficulty to get to the health unities to treat Cutaneous Leishmaniasis?*	Item excluded	
	**SHS26- Com qual freqüência você depende de alguém para te acompanhar nas consultas do tratamento da leishmaniose cutânea?** **How often do you depend on someone else to accompany you to your medical appointments to treat Cutaneous Leishmaniasis?*	Item not changed	**SHS26- Com qual freqüência você depende de alguém para te acompanhar nas consultas do tratamento da leishmaniose cutânea?** **How often do you depend on someone else to accompany you to your medical appointments to treat Cutaneous Leishmaniasis?*
	**SHS27- Com qual freqüência você já dependeu dos serviços de saúde para fornecimento de material ou para ajuda na troca de curativos das feridas?** **How often have you relied on health services to provide you with supplies or to help changing the wound bandages?*	Item not changed	**SHS27- Com qual freqüência você já dependeu dos serviços de saúde para fornecimento de material ou para ajuda na troca de curativos das feridas?** **How often have you relied on health services to provide you with supplies or to help changing the wound bandages?*

**Literal translation of the items into the English language not contemplating any process of cross-cultural adaptation or validation.*
