# Supplementary material for: Development and initial validation of a cutaneous leishmaniasis impact questionnaire
Source: PLoS One. 2018 Aug 30;13(8):e0203378. doi: 10.1371/journal.pone.0203378 (PMC6117079; doi:10.1371/journal.pone.0203378)
Supplement: S2 File — (PDF) [file pone.0203378.s002.pdf]

S2 File. Cutaneous Leishmaniasis Impact Questionnaire (CLIQ).

As questões abaixo avaliam o impacto da leishmaniose cutânea na sua vida. Para cada uma das seguintes questões, por favor, indique a resposta que melhor descreve as suas experiências. Considere desde o início da sua doença até agora ao responder cada pergunta.

Impacto geral da Leishmaniose cutânea	Nada	Levemente	Nem pouco nem muito	Moderadamente	Extremamente
1. A leishmaniose cutânea prejudicou o seu bem-estar geral?	0	1	2	3	4
2. A leishmaniose cutânea interferiu na sua prática de atividade física?	0	1	2	3	4
3. A leishmaniose cutânea afetou de alguma forma a sua capacidade de trabalhar (ou estudar)?					
4. A leishmaniose cutânea aumentou de alguma forma seus gastos com sua saúde?	0	1	2	3	4
5. Você considera que a leishmaniose cutânea prejudicou financeiramente a sua família?	0	1	2	3	4
6. Você está se sentindo isolado das outras pessoas desde que teve a leishmaniose cutânea?	0	1	2	3	4
7. Você já sofreu achando que sua aparência é diferente das pessoas que não têm feridas na pele?	0	1	2	3	4
	Nunca	Quase nunca	Às vezes	Com frequencia	Com muita frequencia
8. Você já teve dificuldade para andar, trocar de roupa ou tomar banho, por causa da(s) ferida(s) na pele?	0	1	2	3	4
9. Você já sentiu dor, ardor, coceira ou incômodo no local da(s) ferida(s) da pele?	0	1	2	3	4
10. Você já ficou nervoso, triste ou com medo por causa da leishmaniose cutânea?	0	1	2	3	4
11. Você já teve sentimento de culpa ou insegurança por causa da leishmaniose cutânea?	0	1	2	3	4
12. Você já se sentiu envergonhado por causa da(s) ferida(s) na pele?					
13. Você já faltou ao trabalho (ou à escola) por causa da leishmaniose cutânea?	0	1	2	3	4
14. Você já teve dificuldade na relação sexual por causa da(s) ferida(s) na pele?	0	1	2	3	4
15. Com que freqüência você precisou pagar alguém para te substituir em atividades do trabalho ou de casa para ir ao serviço de saúde	0	1	2	3	4
16. Você precisou mudar o estilo de se vestir por causa de preconceito de outras pessoas em relação às suas feridas na pele?	0	1	2	3	4
17. Com qual freqüência você está evitando atividades sociais com grupos de pessoas por causa da leishmaniose cutânea?	0	1	2	3	4
18. Com qual freqüência você depende de alguém para te acompanhar nas consultas do tratamento da leishmaniose cutânea?	0	1	2	3	4

Percepção sobre o tratamento e serviços de saúde	Muito boa	Boa	Razoável	Ruim	Muito ruim
19. O que você acha da medicação usada para tratar a leishmaniose cutânea?	0	1	2	3	4
20. O que você achou sobre a forma como foi acolhido pelos serviços de saúde em busca do diagnóstico da leishmaniose cutânea?	0	1	2	3	4
21. O que você achou sobre a forma como foi acolhido pelos serviços de saúde em busca do tratamento da leishmaniose cutânea?	0	1	2	3	4
	Nunca	Quase nunca	Às vezes	Com frequencia	Com muita frequencia
22. Com qual freqüência você já passou mal por causa dos remédios que usou para tratar a leishmaniose cutânea?	0	1	2	3	4
23. Com qual freqüência você já dependeu dos serviços de saúde para fornecimento de material ou para ajuda na troca de curativos das feridas?	0	1	2	3	4
	Nada	Levemente	Nem pouco nem muito	Moderadamente	Extremamente
24. O quanto te incomoda a necessidade de procurar os serviços de saúde para o tratamento da leishmaniose cutânea?	0	1	2	3	4
25. O quanto demorou até realizar exames, consultas ou internação relacionados à leishmaniose cutânea?	0	1	2	3	4

The questions below evaluate the impact of Cutaneous Leishmaniasis in your life. For each of the following questions, please, indicate the answer that best describes your experiences. Take under consideration the beginning of your illness until now to answer each question.

*General impact of Cutaneous Leishmaniasis	*Nothing	*Lightly	*Neither a bit nor a lot	*Moderately	*Extremely
*Has Cutaneous Leishmaniasis affected your overall well-being?	0	1	2	3	4
*Has Cutaneous Leishmaniasis interfered with your physical activities?	0	1	2	3	4
*Has Cutaneous Leishmaniasis somehow affected your ability to work (or study)?	0	1	2	3	4
*Has Cutaneous Leishmaniasis somehow increased your health expenses?	0	1	2	3	4
*Do you consider that Cutaneous Leishmaniasis has financially damaged you family´s budget?	0	1	2	3	4
*Do you feel isolated from others since you got Cutaneous Leishmaniasis?	0	1	2	3	4
*Have you suffered thinking that your appearance is different from people who don´t have wounds on their skin?	0	1	2	3	4
	*Never	*Almost never	*Sometimes	*Often	*Frequently
*Have you had difficulty walking, changing clothes or bathing because of the wound (s) on your skin?	0	1	2	3	4
*Have you felt pain, burning, itching or discomfort at the site of the skin wound (s)?	0	1	2	3	4
*Have you ever felt nervous, sad or scared because of Cutaneous Leishmaniasis?	0	1	2	3	4
*Have you ever felt guilty or insecure about Cutaneous Leishmaniasis?	0	1	2	3	4
*Have you ever felt embarrassed because of the skin wound(s)?	0	1	2	3	4
*Have you ever missed work (or school) because of Cutaneous Leishmaniasis?	0	1	2	3	4
*Have you ever had difficulty during sexual intercourse because of the skin wound(s)?	0	1	2	3	4
*How often have you had to pay someone to replace you in work or home activities so you could go get health service?	0	1	2	3	4
* Did you have to change the style of dressing because of other people's prejudices about their skin wounds?	0	1	2	3	4
*How often have you avoided social activities with groups of people because of Cutaneous Leishmaniasis?	0	1	2	3	4
*How often do you depend on someone else to accompany you to your medical appointments to treat Cutaneous Leishmaniasis?	0	1	2	3	4

* Perception about health services and treatment	*Very good	*Good	*Middling	*Bad	*Very bad
*What do you think about the medication you used to treat Cutaneous Leishmaniasis?	0	1	2	3	4
*What did you think about how you were welcomed by the health services when you were seeking diagnosis of Cutaneous Leishmaniasis?	0	1	2	3	4
*What did you think about how you were welcomed by the health services when you were seeeking treatment for Cutaneous Leishmaniasis?	0	1	2	3	4
	*Never	*Almost never	*Sometimes	*Often	*Frequently
*How often have you felt sick because of the medications you took to treat Cutaneous Leishmaniasis?	0	1	2	3	4
*How often have you relied on health services to provide you with supplies or to help changing the wound bandages?	0	1	2	3	4
	*Nothing	*Lightly	*Neither a bit nor a lot	*Moderately	*Extremely
* How much do you care about the need to seek health services for the treatment of cutaneous leishmaniasis?	0	1	2	3	4
*How long has it taken to get the tests done, medical appointments or hospitalizations related to Cutaneous Leishmaniasis?	0	1	2	3	4

Literal translation of the items into the English language not contemplating any process of cross-cultural adaptation or validation.
